# Effect of CAD/CAM Position and Thickness of Ultra-Translucent Multilayered Zirconia on Color Aspects

**DOI:** 10.1155/2023/5000800

**Published:** 2023-08-19

**Authors:** Palanupap Pongtongkham, Nathawat Pleumsamran, Kullapop Suttiat

**Affiliations:** Department of Prosthodontics, Faculty of Dentistry, Chiang Mai University, Chiang Mai 50200, Thailand

## Abstract

**Background:**

Ultra-translucent multilayered zirconia restorations fabricated using computer-aided design and computer-aided manufacturing (CAD/CAM) technology have recently gained popularity. Their esthetic appeal is crucially dependent on the color accuracy, influenced by prosthesis thickness and multilayer composition due to CAD/CAM milling positions. This study comprehensively investigated how these two factors impacted color accuracy, thereby enhancing our understanding of color outcomes.

**Materials and Methods:**

One hundred monolithic multilayer zirconia specimens with 10 × 10 mm square shape were milled in four different positions and five different thicknesses (1.0, 1.5, 2.0, 2.5, and 3.0 mm). The specimens were placed on an A3 shade resin composite substrate, and CIELAB values (*L* ^*∗*^, *a* ^*∗*^, and *b* ^*∗*^) were measured using a spectrophotometer. Delta *E* (*ΔE*) values were calculated to quantify the color differences between the specimens and the A3 VITA classical shade tab and compared with the perceptibility and acceptability thresholds of *ΔE* = 1.2 and 2.7, respectively. Pearson correlation, two-way ANOVA, and Tukey multiple comparisons (*α* = 0.05) were performed.

**Results:**

The proportion of the dentin layer was positively correlated with the *a* ^*∗*^ and *b* ^*∗*^ values, while specimen thickness was positively correlated with the *a* ^*∗*^ value and negatively correlated with the *L* ^*∗*^ and *b* ^*∗*^ values. Significant difference in *ΔE* value due to different CAD/CAM positions was not observed within the same specimen thickness. Perceptible color differences were observed in specimens with thicknesses greater than 1 mm, while specimens with 1 mm thickness fell within the clinically acceptable range. Highest *ΔE* value was found in the specimen with 1 mm thickness.

**Conclusions:**

Different compositions of multilayers in the final restoration due to different CAD/CAM positions do not significantly affect the color appearance of ultra-translucent multilayer zirconia, with color only influenced by specimen thickness.

## 1. Introduction

Fabrication of the fixed dental prostheses from zirconia (ZrO_2_) has recently gained popularity as an alternative to traditional metal-based restorations [1]. Zirconia offers many advantages including an acceptable natural appearance, excellent biocompatibility, impressive strength, and high resistance to wear and corrosion in the oral environment [2]. However, material opacity and grayish-white color are major inherent drawbacks that restrict the application of zirconia in cases with high-esthetic demand [3]. To overcome these issues, monolithic zirconia with high translucency and multiple layers with gradient shade referred to as ultra-translucent zirconia (5Y-PSZ) has been recommended clinically [4]. Blending these esthetic advancements with the remarkable mechanical properties of zirconia produces ultra-translucent zirconia which has emerged as the preferred material for production of fixed dental prostheses. In the anterior region where esthetics are paramount, using ultra-translucent zirconia facilitated by computer-aided design and computer-aided manufacturing (CAD/CAM) technologies is gaining increasing recognition [5].

Translucency and color of the final restoration are the two main factors that determine the esthetics and natural appearance of the dental prostheses [6], while the substrate is also pivotal for the final aspect of the restoration regardless of the type of restorative material and should always be considered [7]. The color of ultra-translucent multilayered zirconia fixed dental prostheses is also significantly influenced by intrinsic factors such as material microstructure and chemical composition, while clinical and laboratory-related factors such as the original color of the abutment stump and type and color of the dental cement, including the restoration design and the milling process, also play a major role in the color and appearance of the final restoration [8].

Extensive research has focused on the relationship between material translucency, color, and dental prostheses [9–12]. The CIELAB color system is often used for the assessment of color differences between two objects. Visual thresholds play a critical role in quality control and aid in the evaluation of color disparities in dental materials, as well as the interpretation of both clinical and in vitro research findings [13]. CAD/CAM multilayered zirconia restorations may exhibit color deviations from the designated shade tabs that exceed the color perceptibility threshold [14]. To address this problem, controlling the thickness and CAD/CAM position during the planning and laboratory processes are crucial. These adjustments optimize the color accuracy and contribute to improved esthetic restoration outcomes.

The importance of prosthesis thickness and CAD/CAM position to maximize the color accuracy of dental prostheses is well-recognized but research that specifically investigates the impact of prosthesis thickness on color accuracy is lacking [15, 16]. No previous studies have addressed the influence of CAD/CAM positions on the final restoration color or explored the combined effects of both factors on color accuracy. Therefore, this study investigated the influence of prosthesis thickness and CAD/CAM positions on color accuracy and determined the optimal values of these factors to achieve a final color that matched a standard shade. As the null hypothesis, this study posited that the color accuracy of ultra-translucent monolithic multilayer zirconia was independent of the milled prosthesis thickness and CAD/CAM positions.

## 2. Materials and Methods

### 2.1. Specimens Preparation

**Figure 1:**
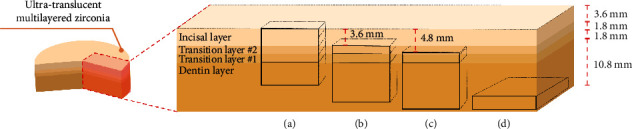
Different milling positions of multilayer zirconia. The specimens were milled at different vertical positions. (a) Position A, the upper edge of the blank; (b) Position B, the center of the blank; and (c) Position C, the lower edge of the blank. By contrast, (d) Position D served as the control group for the CAD/CAM position factor, featuring a horizontal orientation within the dentin layer.

Ultra-translucent multilayered zirconia specimens (Cercon® xt ML; Dentsply Sirona, Bensheim, Germany) with an A3 shade were prepared with dimensions of 10 × 10 mm in four different milling positions (A, B, C, and D) and five different thicknesses (1.0, 1.5, 2.0, 2.5, and 3.0 mm) using CAD/CAM technology (Cerec5 software version 5.x, Inlab MC X5; Dentsply Sirona, Bensheim, Germany), with five samples (*n* = 5) for each milling position and thickness combination. To compensate for sintering shrinkage, the specimens were initially prepared 24.11% larger than the desired final dimensions. The CAD/CAM milling positions are illustrated in Figure 1(a)–1(d), with layer compositions for different CAD/CAM milling positions shown in Table 1.

All specimens were sintered at 1,500°C for 170 min using an Inlab Profile (Dentsply Sirona, Bensheim, Germany), following the manufacturer's recommended guidelines. The sintered specimens were sequentially polished with 600, 800, 1,000, and 1,200-grit silicon–carbide sandpapers using a polishing machine (Mopao 160E, LaiZhou Weiyi Experimental Machinery Manufacture, Shandong, China) to standardize the surface roughness and obtain the predetermined final dimensions [17]. The surface roughness (Ra) of each specimen was assessed using a profilometer (SJ-310 Surface Roughness Measuring tester; Mitutoyo, Kawasaki, Japan) set with cutoff length 0.8 mm, stylus speed 0.5 mm/s, and sampling length 5.0 mm to ensure uniformity among the samples. Specimen dimensions were measured with a digital caliper (Digimatic caliper; Mitutoyo, Kawasaki, Japan). The specimens were sonicated in distilled water at room temperature for 10 min to remove any remnant particles and debris [18].

### 2.2. Substrate Background Preparation

**Figure 2:**
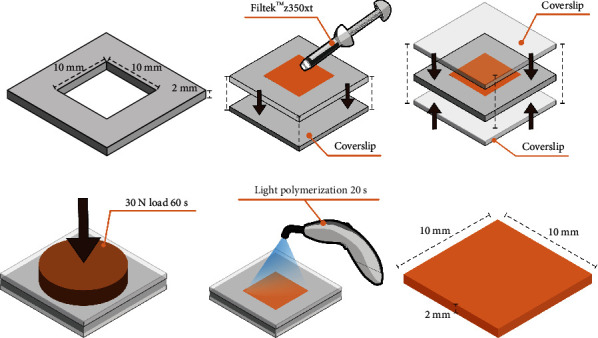
Fabricating process of the 10 × 10× 2 mm substrate background using A3 body shade resin (a–f).

To simulate the effect of the dentin structural component on the appearance of the final restoration, a 10 × 10 × 2 mm A3 body shade resin composite (Filtek™ Z350XT; 3 M ESPE, St. Paul, MN, USA) was used as a background during color measurement, following the method used in a previous study [19]. The preparation process is presented in Figure 2(a)–2(f).

### 2.3. Measurement of CIELAB Values

The color attributes (*L* ^*∗*^, *a* ^*∗*^, and *b* ^*∗*^ values) of multilayer translucent zirconia specimens with different CAD/CAM positions and thicknesses were measured in the CIELAB color system using a spectrophotometer (Agilent Cary 5,000 UV–VisNIR spectrophotometer; Agilent Technologies, Inc, Santa Clara, CA, USA) in accordance with the ISO standard [20]. Measurements were taken at wavelengths between 360 and 830 nm with an interval of 1 nm and a 0°/45° optical geometry. Data were recorded as %*R* (reflectance). The spectrophotometer was recalibrated after every 10 specimens using a white standard (Spectralon® Diffuse Reflectance Standards; Labsphere, Inc., North Sutton, NH, USA) [15].

**Figure 3:**
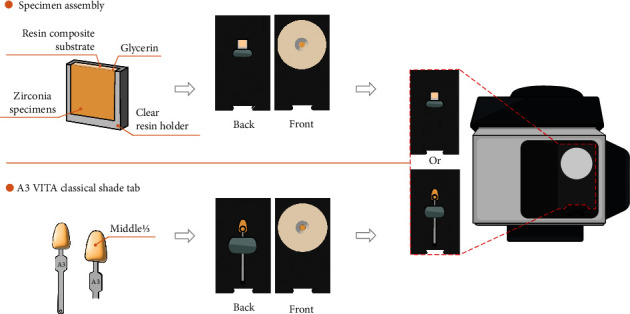
Spectrophotometric measurement process. The specimen assembly and A3 VITA classical shade tab were attached to the specimen holder, with specimen color measured in the CIELAB system by the spectrophotometer.

Each zirconia specimen was placed on the resin composite substrate with a drop of glycerin between the surfaces to reduce the light refraction [21]. The specimen and the resin composite substrate (*L* ^*∗*^ = 91.859, *a* ^*∗*^ = −0.08, and *b* ^*∗*^ = 1.194) were mounted in a clear resin holder. The assembly was attached to the diaphragm of the spectrophotometer. The color was measured in triplicate in the CIELAB system and reported as an average value. Color attributes of the reference A3 VITA classical shade tab were measured at the middle (1/3) in triplicate [22], resulting in average color attributes of *L* ^*∗*^ = 91.483, *a* ^*∗*^ = 0.129, and *b* ^*∗*^ = 1.462.  Figure 3 presents the spectrophotometric measurement process.

### 2.4. Data and Statistical Analysis

The lightness (*L* ^*∗*^), a green–red coordinate (*a* ^*∗*^), and a blue–yellow coordinate (*b* ^*∗*^) in the CIELAB system were reported. Color differences (*ΔE*) between the specimen and the A3 VITA classical shade tab were calculated using the color difference formula in the CIELAB system as follows [23]:

(1) $$\begin{array}{c} \Delta E={\left\{{\left(\Delta L^{*}\right)}^{2}+{\left(\Delta a^{*}\right)}^{2}+{\left(\Delta b^{*}\right)}^{2}\right\}}^{1/2}, \end{array}$$

where *L* ^*∗*^, *a* ^*∗*^, and *b* ^*∗*^ are lightness, a greenred coordinate, and a blue–yellow coordinate in the CIELAB system, respectively. The total difference between the two colors is represented by *ΔE*. To evaluate color differences, the threshold for acceptability and perceptibility were assumed as *ΔE* = 2.7 and *ΔE* = 1.2, respectively [20].

Data from all experiments were statistically analyzed using the Shapiro–Wilk test to assess the normal distribution and Levene's test for homogeneity of variance. Pearson correlation was employed to analyze the correlations between color attributes (*L* ^*∗*^, *a* ^*∗*^, *b* ^*∗*^, and *ΔE*) and thickness, as well as correlations between color attributes and CAD/CAM positions. The results of color difference (*ΔE*) were analyzed by two-way ANOVA. Multiple comparisons were evaluated using Tukey's honestly significant difference test. All statistical tests were conducted at a confidence level of 95% (*p* < 0.05) and a test power of 80%.

## 3. Results

**Figure 4:**
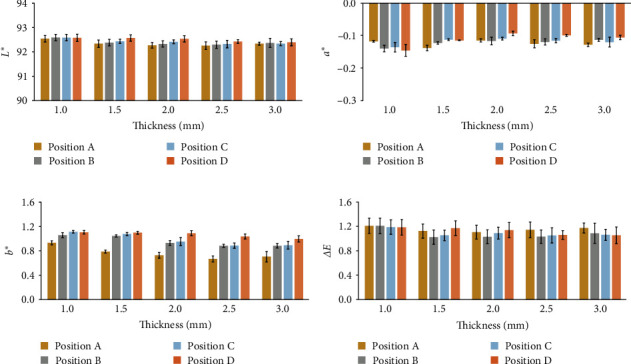
Mean values and standard deviations of the CIELAB color attributes for each specimen group. (a) Lightness (*L* ^*∗*^), ranging from 0 (perfect black) to 100 (perfect reflecting diffuser). (b) Green–red coordinate (*a* ^*∗*^), where positive values denote red and negative values signify green. (c) Blue–yellow coordinate (*b* ^*∗*^), with positive values indicating yellow and negative values representing blue. (d) Color difference (*ΔE*).

Mean and standard deviation of the CIELAB color attributes (*L* ^*∗*^, *a* ^*∗*^, *b* ^*∗*^, and *ΔE*) for each specimen group are presented in Figure 4.  Figure 4(a) illustrates the lightness (*L* ^*∗*^) values, with specimens exhibiting higher brightness (mean *L* ^*∗*^ values between 92.255 and 92.588) compared to the A3 VITA classical shade (*L* ^*∗*^ = 91.483).  Figure 4(b) displays *a* ^*∗*^ values, with specimens showing less redness (mean *a* ^*∗*^ values from −0.137 to −0.093) relative to the A3 VITA classical shade (*a* ^*∗*^ = 0.129).  Figure 4(c) presents the *b* ^*∗*^ values, with specimens revealing less yellowness (mean *b* ^*∗*^ values between 0.664 and 1.112) compared to the A3 VITA classical shade (*b* ^*∗*^ = 1.462), while Figure 4(d) showcases color differences (*ΔE*) between the specimens and the A3 VITA classical shade.

Pearson correlation analyses were conducted to examine the relationship between specimen thickness and color attributes, stratified by CAD/CAM positions (Table 2), and the correlation between CAD/CAM positions (considering the proportion of the dentin layer) and color attributes in the CIELAB system, stratified by specimen thickness (Table 3). The results revealed that specimen thickness was positively correlated with *a* ^*∗*^ values but negatively correlated with *L* ^*∗*^, *b* ^*∗*^, and *ΔE* values. The CAD/CAM positions (considering the proportion of the dentin layer) were positively correlated with *a* ^*∗*^ and *b* ^*∗*^ values, while 1 mm thick specimens showed a negative correlation with the *a* ^*∗*^ value.

The analysis further demonstrated that as specimen thickness increased, the *ΔE* value decreased, with the zirconia specimen color more closely resembling the A3 VITA classical shade. A lower CAD/CAM position, corresponding to a higher proportion of the dentin layer, led to increased *a* ^*∗*^ and *b* ^*∗*^ values but showed no correlation with the *ΔE* value.

**Figure 5:**
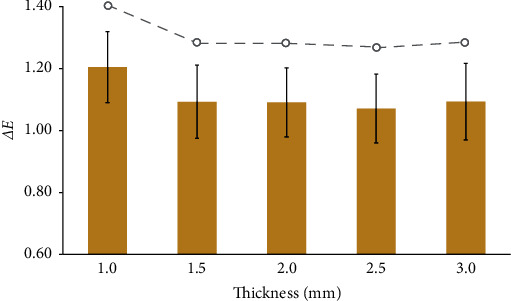
Mean values and standard deviations of color differences (*ΔE*) for specimens with varying thicknesses.

Two-way ANOVA analysis revealed no interaction between specimen thickness and CAD/CAM positions, with only the thickness affecting the *ΔE* value. As shown in Figure 5, the *ΔE* value tended to decrease as the thickness of the specimens increased, consistent with the Pearson correlation analysis. Notably, perceptible color differences (*ΔE* ≤ 1.2) were observed in specimens with thickness greater than 1 mm, while the 1 mm thickness group exhibited color differences that fell within the clinically acceptable range (1.2 < *ΔE* ≤ 2.7).

Regardless of the CAD/CAM positions, *ΔE* values for the 1.5, 2.0, 2.5, and 3.0 mm specimens were not significantly different (*p* > 0.05). However, significance was observed in the group with 1 mm thickness (*p* ≤ 0.05). *ΔE* values obtained from specimens with different CAD/CAM positions but the same thickness were also not significantly different (*p* > 0.05).

## 4. Discussion

This study investigated the influence of thickness and CAD/CAM positions on the color accuracy of ultra-translucent multilayered zirconia. The null hypothesis suggested that color accuracy would be independent of both factors. However, results demonstrated significant differences in *ΔE* values related to zirconia specimen thickness when compared to the A3 VITA classical shade, indicating that color accuracy primarily depended on thickness, while CAD/CAM position had no significant impact. Consequently, the null hypothesis was rejected.

Color attributes (*L* ^*∗*^, *a* ^*∗*^, and *b* ^*∗*^ values) of zirconia ceramic are brand dependent. Various factors influence these attributes including microstructure and composition differences among manufacturers, as well as the shade of zirconia, background substrate, cement, and study design [8, 10]. These factors contribute to the unique optical properties and color attributes of zirconia ceramic. The *L* ^*∗*^ value, representing brightness, exhibited a negative correlation with thickness due to its association with incident light reflection. As zirconia thickness increased, scattering and absorption of light increased within the material, leading to a decrease in the *L* ^*∗*^ value [24, 25]. Furthermore, the *L* ^*∗*^ value tended to increase with lower positioning of the zirconia blank, associated with less translucency [26]. This reduced translucency was due to decreased transmittance of light through the zirconia material, which in turn resulted in increased reflection of the incident light [3, 27].

The chroma (*a* ^*∗*^ and *b* ^*∗*^ values) of the zirconia blank in various regions were different due to varying concentrations of colorant. Manufacturers often add metal oxides, such as ferric oxide (Fe_2_O_3_), to induce a yellow hue and create a natural shade gradient [28]. In this study, as the thickness increased, the *a* ^*∗*^ value exhibited an upward trend, while the *b* ^*∗*^ value demonstrated a decrease because an increase in zirconia thickness resulted in greater light absorption, leading to changes in color saturation [15]. The *b* ^*∗*^ value was reported as more sensitive to changes in thickness than the *a* ^*∗*^ value [29]. The findings of this study concurred with the previous investigations [29–31].

Results also revealed that the test group specimens exhibited higher *L* ^*∗*^ values but lower *a* ^*∗*^ and *b* ^*∗*^ values compared to the A3 VITA classical shade, suggesting that monolithic multilayered zirconia was designed to support the additive staining [32]. As a result, the *L* ^*∗*^ value decreased while the *a* ^*∗*^ and *b* ^*∗*^ values increased after staining [33], bringing the restoration color closer to the A3 VITA classical shade tab.

Color difference can be interpreted using visual color thresholds [2]. In clinical applications, the thresholds of 50% : 50% perceptibility (where 50% of observers notice a color difference while the other 50% do not) and 50% : 50% acceptability (where 50% of observers accept a color difference while the other 50% do not) are utilized [13, 34]. Perceptible *ΔE* thresholds in various studies ranged from 1.0 to 3.7, while acceptable *ΔE* thresholds ranged from 1.7 to 6.8 [35]. Employing different visual color thresholds can lead to variations in the interpretation of research results [36]; however, no consensus exists on the visual color threshold. Consequently, this study adopted thresholds as per the requirements of ISO/TR 28,642 Dentistry-Guidance on color measurement as 50% : 50% perceptibility (at *ΔE* ≤ 1.2) and 50% : 50% acceptability (at *ΔE* ≤ 2.7) [20]. Findings indicated that a thickness of 1 mm was clinically acceptable (1.2 < *ΔE* ≤ 2.7), while a thickness of ≥1.5 mm was undetectable (*ΔE* ≤ 1.2). This outcome was attributed to the influence of translucency on color saturation and masking ability from the background color [30], consistent with previous research.

Kang et al. [23] assessed the accuracy of the final colors of three different types of high-translucency monolithic zirconia with varying thicknesses placed on three distinct color backgrounds (gray, transparent, and A2). They found that both thickness (main factor) and type of zirconia blocks influenced color accuracy of high-translucency zirconia, while Tabatabaian et al. [16] reported that different zirconia brands did not affect the final color when the brands had similar translucency and underwent the same coloring process.

Kim et al. [29] investigated the effect of thickness reduction on the color and translucency of monolithic zirconia. Their findings revealed that reducing the thickness of zirconia from 2 to 1 mm led to an increase in the translucency parameter (TP) as well as a more noticeable reddish and bluish appearance, while Kang et al. [15] observed that increasing zirconia thickness from 0.5 to 2 mm resulted in a decrease in the *ΔE* value, thereby enhancing color accuracy.

Several studies have reported that the final color of highly translucent zirconia is influenced by the background color. For instance, Hsu et al. [37] and Tabatabaian et al. [38] indicated that different abutment colors provided different TP and *ΔE* values as well as color attributes. Consistent with these findings, various research has suggested a minimum thickness of 1 mm for color accuracy on a light color background and a minimum thickness of 1.5 mm for the optimal masking ability [15, 39, 40], consistent with the results of this study which determined distinct color attributes at 1 mm thickness compared to other thicknesses.

Statistically, the *ΔE* values of CAD/CAM were not significantly different for each position. However, average *ΔE* data in Figure 4(d) showed that the bottom position provided color perceptibility closest to the A3 VITA classical shade (*ΔE* ≤ 1.2), resulting from the color of the dentin layer (highest *a* ^*∗*^ and *b* ^*∗*^ values). Furthermore, low translucency allowed the CAD/CAM position closer to the bottom of the blank to anticipate masking outcomes in a dark background [26]. Nevertheless, this must be considered in conjunction with patient satisfaction regarding esthetics, tooth morphology, and occlusion concerns [41].

To summarize, this study provides a valuable insights into the impacts of thickness and CAD/CAM positions on the color accuracy of ultra-translucent multilayered zirconia, establishing a foundation for further research and clinical applications. However, some limitations must be acknowledged. This in vitro study did not account for the specific clinical factors such as the color of adjacent natural teeth, soft tissues, and light sources which all influence the final color of zirconia ceramic. Future research should examine these clinical factors to further enhance the practical applicability of these findings.

## 5. Conclusions

The following conclusions were drawn within the limitation of this in vitro study. Thickness influenced the final color of ultra-translucent multilayered zirconia, while the position of CAD/CAM within a blank had no coloring effect. Restorations with 1.5 mm thickness provided optimal color accuracy.

## Figures and Tables

**Table 1:** Layer compositions for different CAD/CAM milling positions.

Position	Layers present
A	Incisal layer, transition layer #2, transition layer #1, dentin layer
B	Transition layer #2, transition layer #1, dentin layer
C	Transition layer #2, transition layer #1, dentin layer
D	Dentin layer

*Note*: Regardless of the selected thickness, all specimens maintained the same layer composition across each milling position.

**Table 2:** Results of the Pearson correlation analyses between specimen thickness and color attributes in the CIELAB system, stratified by CAD/CAM positions.

Color attributes	Position				
	A	B	C	D	Overall
*L* ^*∗*^	−0.44 ^*∗∗*^ (0.026)	−0.45 ^*∗∗*^ (0.024)	−0.62 ^*∗*^ (0.001)	−0.52 ^*∗*^ (0.007)	−0.47 ^*∗*^ (<0.001)
*a* ^*∗*^	−0.10 (0.623)	0.67 ^*∗*^ (<0.001)	0.31 (0.135)	0.67 ^*∗*^ (<0.001)	0.40 ^*∗*^ (<0.001)
*b* ^*∗*^	−0.78 ^*∗*^ (<0.001)	−0.87 ^*∗*^ (<0.001)	−0.87 ^*∗*^ (<0.001)	−0.73 ^*∗*^ (<0.001)	−0.50 ^*∗*^ (<0.001)
*ΔE*	−0.07 (0.743)	−0.25 (0.232)	−0.337 (0.099)	−0.445 ^*∗∗*^ (0.026)	−0.27 ^*∗*^ (0.007)

^*∗*^Correlation significant at the 0.01 level (two-tailed; *p* ≤ 0.01).  ^*∗∗*^Correlation significant at the 0.05 level (two-tailed; *p* ≤ 0.05).

**Table 3:** Results of the Pearson correlation analyses between CAD/CAM positions (considering the proportion of the dentin layer) and color attributes in the CIELAB system, stratified by specimen thickness.

Color attributes	Thickness					
	1 mm	1.5 mm	2 mm	2.5 mm	3 mm	Overall
*L* ^*∗*^	0.11 (0.631)	0.56 ^*∗∗*^ (0.011)	0.66 ^*∗*^ (0.002)	0.41 (0.069)	0.15 (0.528)	0.33 ^*∗*^ (0.001)
*a ^*∗*^*	−0.64 ^*∗*^ (0.002)	0.84 ^*∗*^ (<0.001)	0.63 ^*∗*^ (0.003)	0.71 ^*∗*^ (<0.001)	0.64 ^*∗*^ (0.002)	0.30 ^*∗*^ (0.003)
*b* ^*∗*^	0.87 ^*∗*^ (<0.001)	0.93 ^*∗*^ (<0.001)	0.94 ^*∗*^ (<0.001)	0.96 ^*∗*^ (<0.001)	0.88 ^*∗*^ (<0.001)	0.78 ^*∗*^ (<0.001)
*ΔE*	−0.09 (0.714)	0.09 (0.701)	0.09 (0.704)	−0.30 (0.202)	−0.38 (0.097)	−0.11 (0.271)

^*∗*^Correlation significant at the 0.01 level (two-tailed; *p* ≤ 0.01).  ^*∗∗*^Correlation significant at the 0.05 level (two-tailed; *p* ≤ 0.05).

## Data Availability

Data supporting the conclusions of this research can be obtained from the corresponding author upon reasonable request.
